# Correction to: ERO1α-dependent endoplasmic reticulum-mitochondrial calcium flux contributes to ER stress and mitochondrial permeabilization by procaspase-activating compound-1 (PAC-1)

**DOI:** 10.1038/s41419-025-08108-8

**Published:** 2025-10-21

**Authors:** M. Seervi, P. K. Sobhan, J. Joseph, K. Ann Mathew, T. R. Santhoshkumar

**Affiliations:** Cancer Research Program-1, Rajiv Gandhi Centre for Biotechnology, Thiruvananthapuram, Kerala India

Correction to: *Cell Death & Disease* 10.1038/cddis.2013.502, published online 19 December 2013

We had published two manuscripts in the Cell Death and Disease journal, describing the mechanism of cell death induced by the direct caspase activator compound PAC-1 using cell-based approaches. The work conducted between years 2006–2010 primarily in two cell models, MEF and MCF7. The first manuscript was titled “Essential requirement of cytochrome c release for caspase activation by procaspase-activating compound defined by cellular models. Cell Death Dis. 2011 2. The second paper describing the mechanism of action of PAC-1 on ERO1-dependent signalling was published in 2013, “ERO1α-dependent endoplasmic reticulum-mitochondrial calcium flux contributes to ER stress and mitochondrial permeabilization by procaspase-activating compound-1 (PAC-1). Cell Death Dis. 2013 Dec 19;4(12).

Subsequent to a very recent Pubpeer alert, it came to our notice that a western blot of housekeeping gene Hsc70 in the figure 2b panel of our 2013 article (Cell Death Dis. 2013 Dec 19;4(12):e968) was accidentally replaced with an earlier used beta actin blot in a 2011 article.

**Incorrect Figure 2b** 
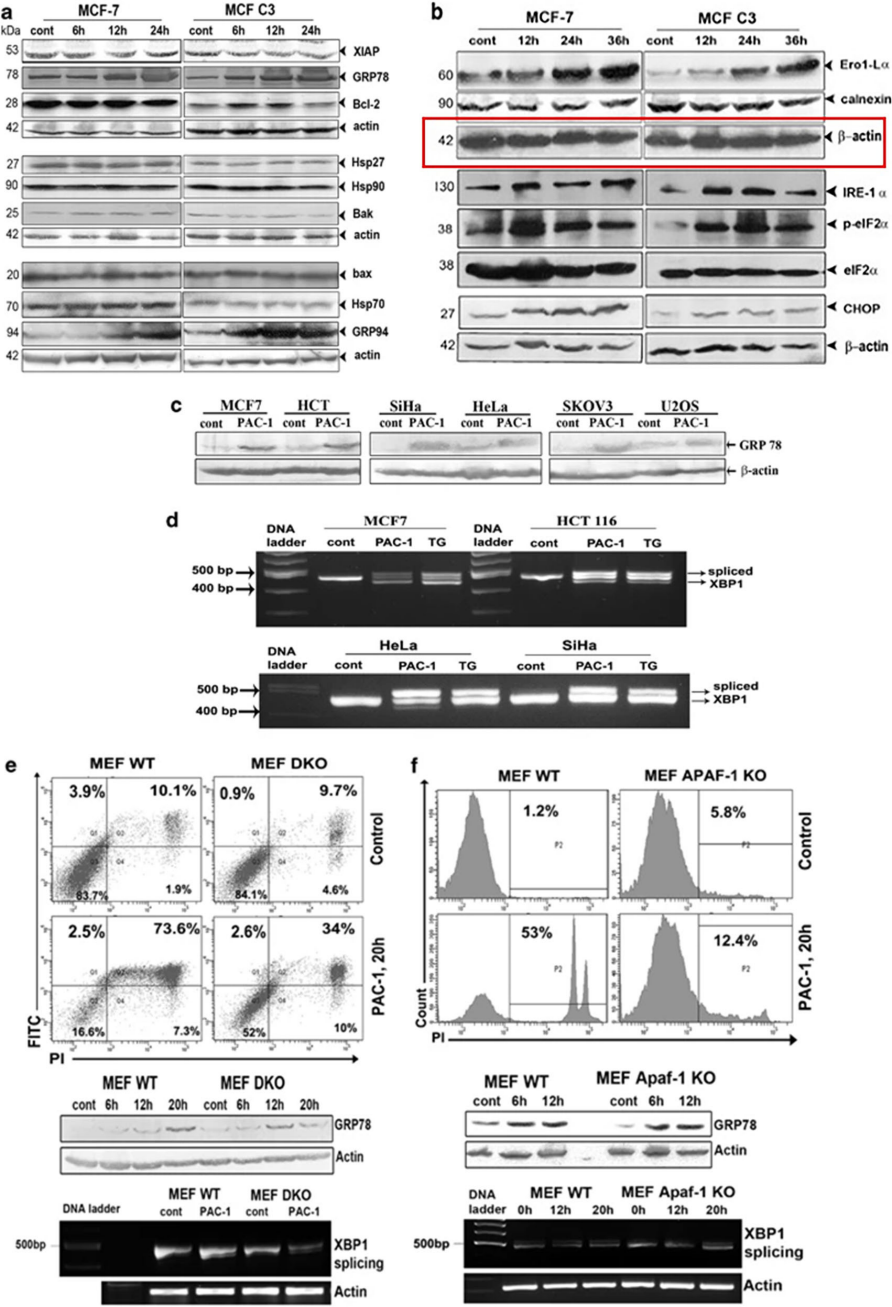


**Correct Figure 2b** 
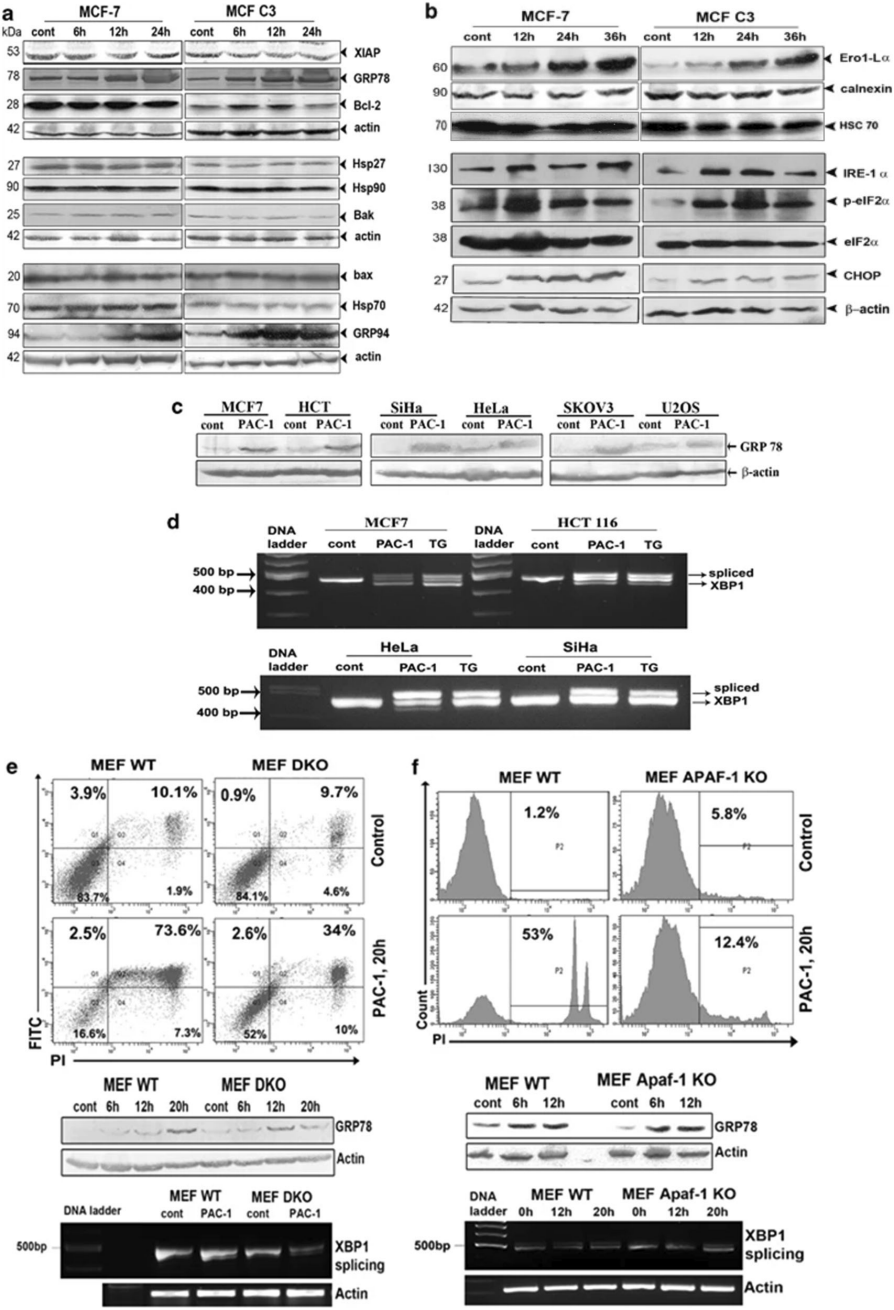


The original article has been corrected.

## Supplementary information

Original Data (Fig 2b -2013)

