# Supplementary figures and images for: Correction to: ERO1α-dependent endoplasmic reticulum-mitochondrial calcium flux contributes to ER stress and mitochondrial permeabilization by procaspase-activating compound-1 (PAC-1)
Source: Cell Death Dis. 2025 Oct 21;16(1):740. doi: 10.1038/s41419-025-08108-8 (PMC12540647; doi:10.1038/s41419-025-08108-8)

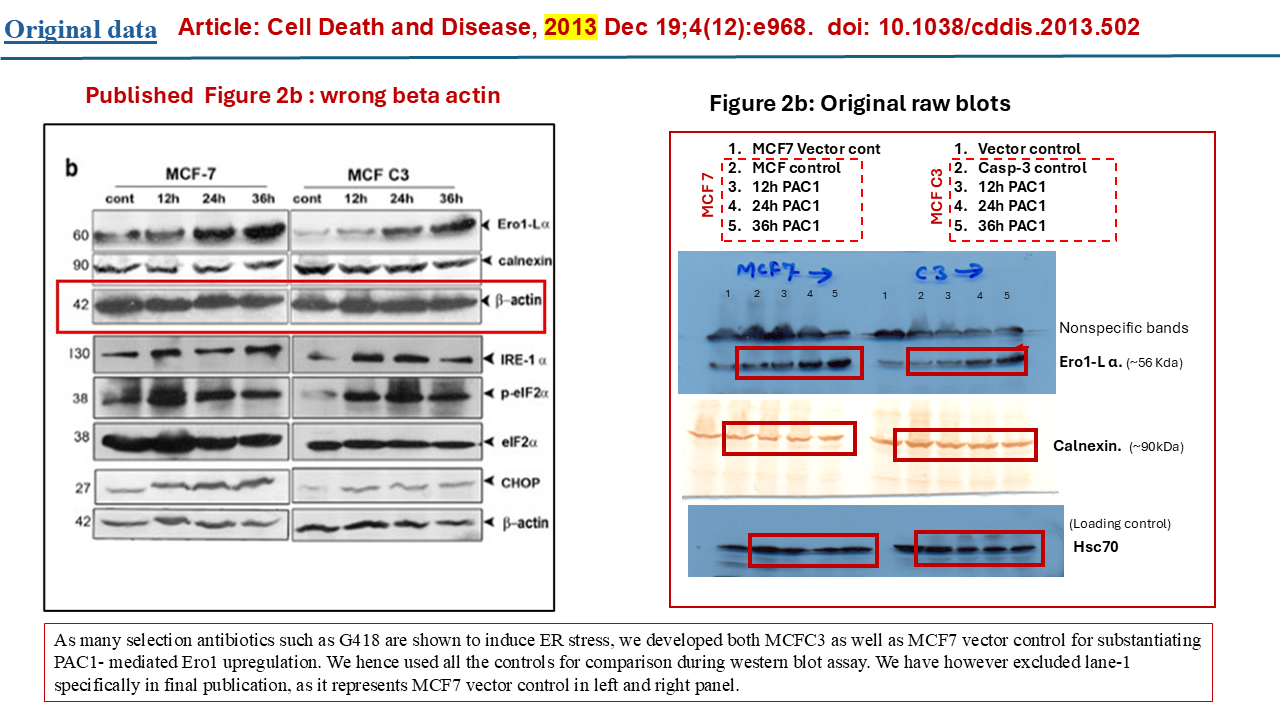

Supplement: Supplementary file 1 — Original Data (Fig 2b -2013) [file 41419_2025_8108_MOESM1_ESM.tif]
